# Comparison of the Effects of Pectoral Nerve Block and Local Infiltration Anesthesia on Postoperative Pain for Breast Reduction Surgery: A Prospective Observational Study

**DOI:** 10.5152/eurasianjmed.2021.20111

**Published:** 2021-06

**Authors:** Orcun Sercan, Arzu Karaveli, Sadik Ozmen, Asim Uslu

**Affiliations:** 1 Department of Anesthesiology and Reanimation, University of Health Sciences, Antalya Training and Research Hospital, Antalya, Turkey; 2 Department of Plastic and Reconstructive Surgery, University of Health Sciences, Antalya Training and Research Hospital, Antalya, Turkey

**Keywords:** nerve block, mammaplasty, postoperative pain

## Abstract

**Objective:**

To evaluate the effects of the Pecs II block on postoperative pain in patients undergoing breast reduction surgery.

**Materials and Methods:**

This prospective, comparative, and observational study was conducted with 53 patients, with American Society of Anesthesiologists I-II, between the ages of 18 and 65, and undergoing bilateral breast reduction surgery. The patients were divided into two groups: Pecs II block with general anesthesia (Pecs group; n = 26) and local infiltration anesthesia with general anesthesia (control group; n = 27). The patients’ demographic data, duration of surgery and anesthesia, hemodynamic parameters, perioperative analgesia requirements, postoperative visual analog scale (VAS) scores (at zero, one, three, six, nine, and 12 hours postoperative), the number of patients who needed analgesia at least once, the length of the hospital stay, and block-related complications were recorded.

**Results:**

There was no statistical difference in terms of the duration of surgery and anesthesia and hemodynamic parameters. Intraoperative total fentanyl consumption (128.85 ± 25.19 mcg in the Pecs group and 227.77 ± 44.58 mcg in the control group; *P* < .001) and postoperative analgesic requirement were significantly lower in the Pecs group (*P* < .001). The number of patients who needed analgesia at least once in the Pecs group was four (15.3%). Postoperative VAS scores were significantly lower (*P* < .001) and the length of the hospital stay was significantly shorter in the Pecs group (*P* < .001). No block-related complications were observed.

**Conclusion:**

Pecs II block with general anesthesia may significantly contribute to reducing intraoperative and postoperative analgesia requirements and provide long-lasting and more effective postoperative pain in breast reduction surgery.

## Introduction

Breast surgery is one of the most commonly performed procedures in hospitals and one of the most common causes of postoperative pain. Studies have shown that the incidence of postoperative pain may vary between 12%–57% after breast cancer surgery^1^ and 21%–50% after non-cancer breast surger.^2^ It has also been reported that untreated postoperative pain may lead to chronic pain syndrome and consequently increase the length of the hospital stay, need for analgesia, and in-hospital costs.^3^

Thoracic paravertebral block, interscalene brachial plexus block, and/or thoracic epidural analgesia methods are used to eliminate postoperative pain and reduce narcotic analgesic use after breast surgery.^3^–^6^ However, these techniques are not preferred by many anesthetists because of the risk of serious complications and their technical complexity.^7^ In recent years, interfascial plane blocks that can be performed more efficiently and easily such as erector spinae plane block, pectoral nerve (Pecs) block, and superficial serratus plane block have been defined as an alternative to these blocks in the severe postoperative pain management after breast surgery.^8^–^10^

The ultrasound-guided pectoral type 2 (Pecs II) block is an interfacial plane block where local anesthetic is administered between the pectoralis major (PMm) and the pectoralis minor muscle (Pmm) and above the serratus anterior muscle (SAm). It was first described by Blanco in 2012 and defined as an alternative to conventional regional techniques in postoperative pain management after breast surgery.^10^ A systematic review and meta-analysis evaluating the analgesic efficacy of the Pecs block in breast surgery demonstrated that the Pecs II block significantly improved quality of analgesia, decreased opioid consumption, and provided similar analgesic efficacy as the thoracic paravertebral block.^7^,^11^

In this study, we aimed to evaluate the effects of the Pecs II block on postoperative pain in patients undergoing breast reduction surgery. The difference in postoperative visual analog scale (VAS) scores in patients who had Pecs II block combined with general anesthesia, compared with those having local infiltration anesthesia combined with general anesthesia was the primary outcome of the study.

## Materials and Methods

This prospective, comparative, observational study was approved by the ethics committee (10/09) and was carried out according to the Declaration of Helsinki, and registered in the NCT03857386 clinical research database (clinicaltrials.gov). All the patients were informed, and their consent for general anesthesia was acquired before elective breast reduction surgery between July 2017 and May 2018. Strengthening of Reporting of Observational Studies in Epidemiology (STROBE) guidelines were followed to report this study.^12^

The study included patients aged between 18 and 65, with a body mass index (BMI) ≤ 40 kg/m^2^, having the physical status of I–II as defined by the American Society of Anesthesiologists (ASA), and who were planned to undergo elective bilateral breast reduction surgery. The patients who declined to give written informed consent, were under 18 years of age or over 65 years of age, had ASA physical status of III and above, contraindications of peripheral blocks or local anesthetic infiltration (local infection, coagulopathy, etc.), had a history of allergy against local anesthetics, as well as history of breast surgery, the patients who had a nerve block and chronic pain history, and undergoing treatment for psychiatric disorders were excluded from the study. The potential risks, benefits, and complications after the study protocol were fully and thoroughly explained to the patients.

The patients were divided into two groups: Pecs group (Pecs II block with general anesthesia n = 26) and control group (local infiltration anesthesia with general anesthesia; n = 27). Routine preoperative evaluation of patients for elective breast reduction surgery was performed. The patients’ demographic data (age, ASA physical status, and BMI), duration of surgery and anesthesia, preoperative and intraoperative hemodynamic parameters (mean blood pressure [MBP], pulse oximetry [SPO_2_], and heart rate [HR]), intraoperative and postoperative analgesia requirements, postoperative VAS scores, the number of patients who needed analgesia at least once, block-related complications, and length of the hospital stay were recorded.

Data collection and anesthesia management were recorded by a blinded third person (excluding the anesthesiologist who applied the block and surgical team). Hemodynamic parameters were recorded at baseline (before anesthesia induction), five and 15 minutes after induction, before and five minutes after surgical incision, and at the end of surgery.

### Standard Anesthesia Management

This study did not affect standard patient care or the routine anesthesia or surgery protocol used for such patients. On arrival in the operating theatre, an 18 G vascular catheter was placed intravenous (IV) crystalloid fluid infusion was initiated. For anesthesia induction, 2–3 mg/kg IV propofol, 2 mcg/kg IV fentanyl, and 0.6 mg/kg IV rocuronium were used in both groups after premedication with 0.04 mg/kg IV midazolam. Intraoperative ASA basic monitoring protocol was used: electrocardiogram, non-invasive blood pressure, HR, end-tidal carbon dioxide (EtCO_2_), and SpO_2_. The patients were ventilated with the volume-controlled mode, with an EtCO_2_ ranging 35–45 mmHg (Primus Drager, Luebeck, Germany). Anesthesia was maintained with a mixture of 40% air in O_2_ and sevoflurane 2%. To maintain MBP and/or HR values within or 20% lower than baseline values, additional boluses of 0.5 μg/kg IV fentanyl were administered. To ensure adequate muscle relaxation, rocuronium 10 mg IV was re-administered. The patients were given 4 mg IV ondansetron for postoperative nausea and vomiting prophylaxis. A reduction of more than 20% of baseline values and treated with ephedrine 5–10 mg IV and bolus fluid application was defined as hypotension. At the end of the surgery, 0.05 mg/kg neostigmine and 0.02 mg/kg atropine were administered, intravenously. The patients were extubated and transferred to the postanesthesia care unit (PACU) after responding to verbal commands. The duration of anesthesia was defined as the time between the arrival of the patient to the operating room and transfer to the PACU. Pain severity was evaluated with VAS scores, and a sedation degree was evaluated with a Ramsay sedation scale in the PACU.

### Routine Application of Pecs Blocks

Pecs block was applied preoperatively, and all patients were observed under standard monitoring in the regional anesthesia room. After vascular access was provided with an 18 G cannula, IV crystalloid fluid infusion and premedication with 0.04 mg/kg IV midazolam was initiated. To perform the blocks, a Mindray (Shenzhen, China) DC-T6 ultrasound (USG) machine with a 10-MHz linear probe was used. Before all of the blocks, the skin was cleaned with chlorhexidine. The probe was placed below the lateral third of the clavicle. After observing the axillary artery and axillary vein, the probe was moved in the inferolateral direction. PMm, Pmm, SAm, and the fourth rib were ultrasonographically visualized. After fixing the second rib, the third and fourth ribs were also localized by moving and counting the ultrasound probe down and laterally on the chest wall of the patient. The skin puncture point was infiltrated with 2–4 mL of 2% lidocaine after recognition of the appropriate anatomical structures. Then, using the in-plane technique, a 21-gauge 100 mm needle (Stimuplex^®^ A, B. Braun Melsungen AG, Germany) was moved from cephalic to caudal. Two-needle approaches were used to perform the Pecs II block. First, 10 mL of 0.25% bupivacaine was injected between the PMm and Pmm, and then 20 mL of 0.25% bupivacaine was injected between the Pmm and the SAm after confirmation of a negative aspiration for the blood and hydrodissection with 0.5 mL of normal saline. The bilateral Pecs II block was applied, separately. A single clinician (A.K.) performed all the Pecs II blocks. All the patients were observed for 15 minutes before being transferred to the operating theatre. Block-related complications such as intravascular injection, hematoma, and pneumothorax were also recorded.

### Standard Surgical Procedures

The patient was drawn according to the superomedial pedicle and inverted T breast reduction technique when erect, and taken to the operation room. The incision was made according to preoperative drawings. To reduce intraoperative bleeding and decrease postoperative pain, 20 mL of 0.25% bupivacaine and saline solution of diluted epinephrine (1/100,000) were administered at the incision line for breast reduction surgery. However, to reduce the risk of local anesthetic toxicity, only 20 mL of the saline solution of diluted epinephrine (1/100,000) was administered at the incision line when Pecs block was administered. Approximately 10 minutes after the local anesthetic infiltration, the pedicle was de-epithelized. After that, the incision of the breast was made up of the pectoral muscle fascia. The pedicle was preserved and the breast tissue in the superior of this incision was removed in one piece. Hemostasis was performed, and one hemovac drain was placed on each breast. Nipple and areola were suspended to the junction of the inverted T incision with temporary sutures, and the incisions were sutured as two layers. Duration of surgery was defined as the time between the first incision and the end of surgery. The patients (with VAS scores below 3 who were able to eat and mobilized) were discharged from the hospital. The length of the hospital stay was defined as the time from the end of surgery to the discharge of the patient. Every surgery was performed by a single surgeon (A.U.).

### Evaluation of Pain

Postoperative analgesia was evaluated by using VAS score for pain (VAS 0 cm = no pain, VAS 10 cm = most severe pain possible). VAS scores were recorded at zero (obtained within PACU), one, three, six, nine, and 12 hours after surgery by a blinded third person. In our hospital, patients with a postoperative VAS score of 3 cm or above received additional analgesia. If VAS was ≤ 2 cm and/or the patients refused pain medications, analgesia was not administered. For rescue analgesia in the PACU, paracetamol 1 g IV was administered. As rescue analgesia for a postoperative VAS score ≥ 3 cm within the first 24 hours, diclofenac sodium 75 mg IM was administered. If the pain persisted at the same level in following 1 hour period, tramadol 50 mg IV was supplemented. The number of patients who needed analgesia at least once was also recorded from nurse observational forms.

### Primary and Secondary Aims

To evaluate the difference between Pecs II block and local infiltration anesthesia concerning postoperative VAS scores was the primary aim of this study. Perioperative analgesia consumption, length of the hospital stay, and hemodynamic findings between the groups were the secondary aims of this study.

### Statistics Analysis

The demographic characteristics of the patients and their collected data were organized in IBM Statistical Package for the Social Sciences version 25.0 (IBM Corporation, Armonk, NY, USA). Continuous variables were reported as mean ± standard deviation (SD) or median (interquartile range), and categorical data were presented as absolute frequencies and percentages. To test for normal distribution of all continuous data, the Shapiro-Wilk test was used. Pearson’s chi-squared test or Fisher’s exact test were used for the analysis of qualitative variables. Normal distributed continuous data were compared between the groups using the Student t-test. Non-normally distributed continuous data were compared between the groups using the Mann-Whitney U test. Repeated measurements (VAS scores) were compared by repeated-measures analysis of variance, with further paired comparisons at each time interval performed using the t-test. The value of P < .05 was considered statistically significant. The sample size was calculated based on data from previous studies.^13^ The postoperative VAS scores were taken into consideration, and it was determined that at least 20 patients were required per group (type 1 error 0.05 and power 90). Assuming a dropout rate of 20%, at least 24 patients would be needed in each group.

## Results

**Figure 1:**
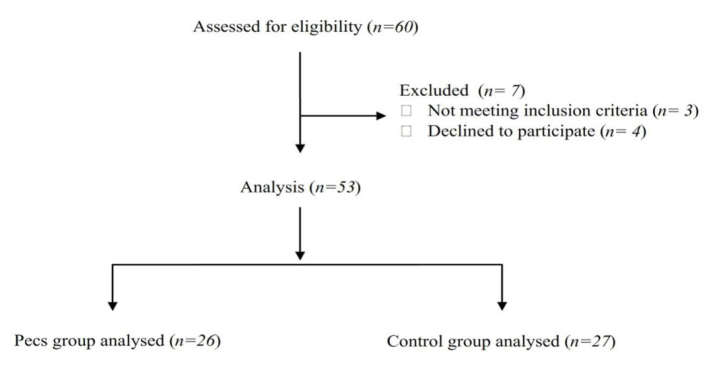
Flow chart of patients.

A total of 60 patients were assessed for study eligibility. Three patients were excluded from the study for not meeting the inclusion criteria, and four patients declined to participate. Therefore, a total of 53 patients were included in the study, 26 in the Pecs group, and 27 in the control group (Figure 1).

Patient characteristics are summarized in Table 1. In both groups, anesthesia and surgery duration were similar (P > .05).

Compared to the Pecs group, intraoperative total fentanyl consumption was significantly higher in the control group, (128.85 ± 25.19 mcg, and 227.77 ± 44.58 mcg, respectively; P < .001).

The preoperative and intraoperative hemodynamic findings of the patients were evaluated, and there was no significant difference between the groups (P > .05) (Table 2).

The number of patients who needed analgesia at least once in the Pecs group was four (15.3%), whereas all the patients in the control group received additional analgesia in the postoperative period (P *<* .001). There was no postoperative tramadol consumption in the Pecs group (P < .001). In the Pecs group, the dose of diclofenac sodium administered was lower; however, there was no significant difference (P = .498) (Table 3).

**Figure 2:**
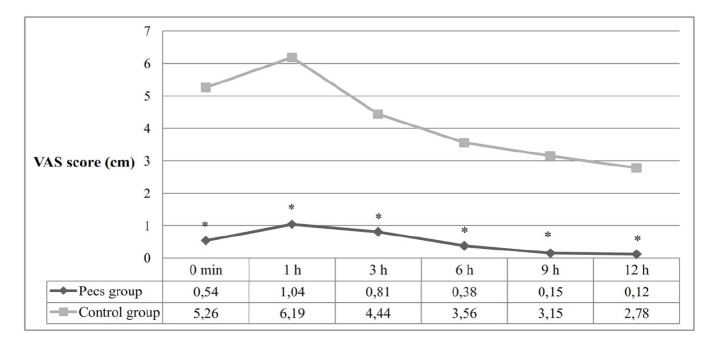
Postoperative Visual Analog Scale scores. (Data are presented as mean. VAS: Visual Analog Scale. The groups were compared by t test after repeated measures ANOVA. *p <0.05, comparisons between the groups.)

VAS scores at zero, one, three, six, nine, and 12 hours after surgery were significantly lower in the Pecs group compared with that in the control group (P < .001) (Figure 2).

The length of the hospital stay was significantly shorter in the Pecs group (1.73 ± 0.45 days in the Pecs group, and 2.30 ± 0.46 days in the control group, P < .001). No block-related complications were observed.

## Discussion

The study showed that the Pecs II block significantly reduced postoperative pain after breast reduction surgery. It was also observed that intraoperative and postoperative analgesic consumption, including the use of intraoperative fentanyl and postoperative paracetamol were much lower in the Pecs group. There was no postoperative tramadol consumption in the Pecs group. Moreover, compared with the control group, the length of the hospital stay was shorter in the Pecs group.

In recent years, Pecs blocks have been used as a part of multimodal analgesia, especially in breast cancer surgery, to reduce postoperative pain, perioperative narcotic consumption, and the need for non-steroid anti-inflammatory drugs, as well as shortening the length of the hospital stay, and avoiding the side effects of narcotics.^14^,^15^ In a prospective randomized controlled study on the effects of Pecs block, it was proved to provide effective analgesia and reduce perioperative narcotic analgesic consumption, including postoperative morphine and intraoperative fentanyl use, and shorten the duration of hospitalization in both hospital and PACU.^14^ A prospective randomized double-blind study evaluating the analgesic efficacy of Pecs block in patients undergoing breast cancer surgery revealed that patients with Pecs block had lower postoperative VAS scores, less need for perioperative analgesics, and shorter duration of hospital stay.^16^

Breast reduction surgery is a common surgical procedure performed by plastic surgeons in the treatment of symptomatic macromastia. Despite high patient satisfaction with long-term outcomes, postoperative pain management is very important in these patients and may result in the need for intravenous narcotic analgesics and long-term hospitalization.^17^ Bilateral breast reduction surgery is associated with significant tissue damage, severe early postoperative pain, and 22% chronic pain incidence one year after the surgery.^18^ To reduce both narcotic analgesic consumption and postoperative pain in patients, a variety of methods such as local anesthetic pumps and preinjection infusion are used.^19^,^20^ In recent years, interfascial plane blocks have been introduced to manage severe postoperative pain after breast reduction surgery.^8^,^9^

The innervation of the breast is complex and provided by many nerves. Motor innervation of the PMm and Pmm is supplied by the lateral and medial pectoral nerves.^21^ Although these nerves are defined as completely motor nerves, they are said to carry proprioceptive and nociceptive fibers.^22^ Cutaneous innervation of the breast is mainly supplied by the lateral and anterior branches of the intercostal nerves. The intercostobrachial nerves are cutaneous sensorial branches of T2, providing innervation of the upper inner arm, axilla, and upper outer quadrant. The anterior branch of the intercostal nerves provides bilateral innervation. However, sensory innervation of the upper quadrant of the breast is supplied by the supraclavicular nerves originating from the upper cervical plexus. Pecs block is a combination of motor and sensory nerve blockade and aims to block intercostobrachial, intercostal III, IV, V, VI, and long thoracic nerves.^14^

To reduce both intraoperative hemorrhage and postoperative pain, plastic surgeons use saline and diluted local anesthetic and epinephrine mixture circumferentially around the breast in breast reduction surgery. However, the efficacy of lidocaine in infiltration solution on postoperative pain remains controversial. Danilla et al.^23^ reported that lidocaine solution added into infiltration solution significantly reduced pain for up to 18 hours after surgery, whereas Christie et al.^17^ reported no significant effect of lidocaine solution on postoperative pain after breast reduction surgery.

Pecs block is administered by anesthesiologists with the guidance of USG before or after induction of general anesthesia. The most important advantage of USG-guided Pecs block is the reduction of possible complications and better visualization of the pleura and paravertebral area.^14^ However, there is a risk of block-related complications such as pneumothorax, hematoma, or intravascular injection. Particular attention should be paid to patients taking oral anticoagulants and antiplatelet drugs.^24^ In our study, we did not observe any block-related complications; however, all our patients were ASA I–II physical status.

Our study had some limitations. First, it was an observational study. Second, we could not record the hourly analgesia consumption of our patients who underwent breast reduction surgery. Finally, we could not evaluate the pain referring to dermatome levels of our patients owing to the surgical site wound dressing and bandage in the postoperative period.

In conclusion, we have demonstrated that the Pecs block has a long-lasting analgesic effect and is more effective than local anesthesia infiltration. Pecs block is an interfascial plane block that is easy to perform and has low rate of complications. Therefore, the Pecs block can be routinely performed in breast surgeries, especially in breast reduction surgery, with or without general anesthesia, which will enable the patients to have a shorter and more comfortable hospital process.

**Main Points**

- Breast surgery is one of the most common causes of postoperative pain.
- PECS block is an interfascial plane blocks that is recently used for postoperative pain after breast surgery.
- It is easy to perform and has low complication rate.
- In the future, PECS block may routinely applied in breast surgery, especially in breast reduction surgery.

## Figures and Tables

**Table 1:** Demographic Characteristics of Patients

	Pecs group (n = 26)	Control group (n = 27)	*P* value
Age (years)	45.5 ± 11.0	43.4 ± 11.4	.492
BMI (kg/m^2^)	30.1 (27.4–34.3)	29.6 (27.3–31.2)	.439
ASA physical status			.916
I	10 (38.5)	10 (37.0)	
II	16 (61.5)	17 (63.0)	
Anesthesia duration (min)	141.5 ± 27.8	142.8 ± 18.3	.831
Surgery duration (min)	120.6 ± 26.9	123.3 ± 19.3	.674

Data are presented as mean ± standard derivation, median (interquartile range) or the number (percentage). P < .05 is statistically significant.

BMI: body mass index, ASA: American Society of Anesthesiologists.

For the analysis of age, duration of anesthesia and surgery, Student t test was used. ASA was compared using Pearson chi-squared test, and BMI was compared by Mann-Whitney U test.

**Table 2:** Hemodynamic Findings of Patients

	Pecs group (n = 26)	Control group (n = 27)	*P* value
HR (before induction), beat/min	78.8 ± 10.0 (74.7–82.8)	81.4 ±8.8 (77.8–84.9)	.323
HR (5 min after induction)	78.4 ± 13.5 (72.9–83.9)	80.1 ± 9.5 (79.3–83.9)	.606
HR (15 min after induction)	77.6 ± 13.3 (72.2–83.0)	76.9 ± 7.9 (73.7–80.0)	.812
HR (before incision)	77.3 ± 12.1 (72.4–82.2)	75.5 ± 7.9 (72.3–78.6)	.373
HR (5 min after incision)	76.5 ± 11.9 (71.6–81.3)	73.3 ± 9.6 (69.5–77.1)	.298
HR (end of the surgery)	76.1 ± 15.7 (69.8–82.5)	75.5 ± 15.9 (69.2–81.8)	.650
MBP (before induction), mmHg	101.7 ± 13.0 (96.4–107.0)	102.3 ± 12.6 (97.2–107.3)	.873
MBP (5 min after induction)	83.0 ± 16.5 (76.3–89.7)	86.7 ± 13.2 (81.5–92.0)	.367
MBP(15 min after induction)	84.7 ± 12.2 (79.8–89.6)	85.5 ± 15.0 (79.6–91.4)	.828
MBP (before incision)	83.6 ± 12.6 (78.4–88.7)	83.2 ± 12.8 (78.1–88.3)	.920
MBP (5 min after incision)	87.1 ± 8.7 (83.5–90.6)	87.5 ± 15.4 (81.4–93.6)	.908
MBP (end of the surgery)	89.1 ± 10.2 (84.9–93.2)	86.6 ± 12.1 (81.8–91.4)	.425
SpO_2_ (before induction), %	98.0 ± 1.0 (97.6–98.5)	98.1 ± 0.9 (97.7–98.5)	.963
SpO_2_ (5 min after induction)	99.1 ± 0.7 (98.8–99.4)	98.8 ± 0.8 (98.5–99.2)	.265
SpO_2_ (15 min after induction)	98.8 ± 0.9 (98.5–99.2)	98.8 ± 0.8 (98.5–99.1)	.874
SpO_2_ (before incision)	99.0 ± 0.9 (98.6–99.4)	98.8 ± 1.0 (98.4–99.2)	.390
SpO_2_(5 min after incision)	98.8 ± 0.8 (98.5–99.2)	98.6 ± 0.8 (98.3–99.0)	.372
SpO_2_ (end of the surgery)	99.1 ± 1.0(98.6–99.5)	99.1 ± 0.9 (98.7–99.5)	.916

Data are presented as mean ± standard derivation (95% confidence interval). P < .05 is statistically significant.

HR: heart rate, MBP: mean blood pressure and SPO_2_: pulse oximetry.

Student t test was used in all the analyses.

**Table 3:** Comparison of Perioperative Analgesic Consumption of Patients

	Pecs group (n = 26)	Control group (n = 27)	*P* value
Need for analgesia, n (%)	4 (15.3)	27 (100)	< .001*
Tramadol (mg), mean ± SD	0	225.9 ± 65.5	< .001*
Paracetamol (gr), mean ± SD	1.0 ± 0.0	2.3 ± 0.6	< .001*
Diclofenac sodium (mg), mean ± SD	75.0 ± 0.0	103.5 ± 37.3	.498

Data are presented as mean ± SD or the number and percentage.

* *P* < .05 is statistically significant.

SD: standard deviation.

Student t test was used in all analyses, except for “needs for analgesia” was compared using Pearson chi-squared test.
